# Patient costs associated with accessing HIV/AIDS care in Malawi

**DOI:** 10.7448/IAS.16.1.18055

**Published:** 2013-03-19

**Authors:** Andrew D Pinto, Monique van Lettow, Beth Rachlis, Adrienne K Chan, Sumeet K Sodhi

**Affiliations:** 1 Department of Family and Community Medicine, St. Michael's Hospital, Toronto, Canada; 2 Centre for Research on Inner City Health, Keenan Research Centre, Li Ka Shing Knowledge Institute, St. Michael's Hospital, Toronto, Canada; 3 Dignitas International, Zomba, Malawi; 4 Dalla Lana School of Public Health, University of Toronto, Toronto, Canada; 5 Division of Infectious Diseases, St. Michael's Hospital, Toronto, Canada; 6 Department of Family and Community Medicine, Faculty of Medicine, University of Toronto, Toronto, Canada; 7 Department of Family and Community Medicine, University Health Network, Toronto Western Hospital, Toronto, Canada

**Keywords:** antiretroviral therapy, highly active, costs and cost analysis, Malawi, HIV, acquired immunodeficiency syndrome

## Abstract

**Introduction:**

The decentralization of HIV services has been shown to improve equity in access to care for the rural poor of sub-Saharan Africa. This study aims to contribute to our understanding of the impact of decentralization on costs borne by patients. Such information is valuable for economic evaluations of anti-retroviral therapy programmes that take a societal perspective. We compared costs reported by patients who received care in an urban centralized programme to those in the same district who received care through rural decentralized care (DC).

**Methods:**

A cross-sectional survey on patient characteristics and costs associated with accessing HIV care was conducted, in May 2010, on 120 patients in centralized care (CC) at a tertiary referral hospital and 120 patients in DC at five rural health centres in Zomba District, Malawi. Differences in costs borne by each group were compared using χ^2^ and *t*-tests, and a regression model was developed to adjust for confounders, using bootstrapping to address skewed cost data.

**Results:**

There was no significant difference between the groups with respect to sex and age. However, there were significant differences in socio-economic status, with higher educational attainment (*p*<0.001), personal income (*p*=0.007) and household income per person (*p*=0.005) in CC. Travel times were similar (*p*=0.65), as was time waiting at the clinic (*p*=0.63) and total time spent seeking care (*p*=0.65). There was a significant difference in travel-related expenses (*p*<0.001) related to the type of travel participants noted that they used. In CC, 60% of participants reported using a mini-bus to reach the clinic; in DC only 4% reported using a mini-bus, and the remainder reported travelling on foot or by bicycle. There were no significant differences between the groups in the amount of lost income reported or other out-of-pocket costs. Approximately 91 Malawi Kwacha (95% confidence intervals: 1–182 MKW) or US$0.59 represents the adjusted difference in total costs per visit between CC and DC.

**Conclusions:**

Even within a system of HIV/AIDS care where patients do not pay to see clinicians or for most medications, they still incur costs. We found that most costs are travel related. This has important implications for poorer patients who live at a distance from health facilities for whom these costs may be significant.

## Introduction

Significant progress has been made against the HIV pandemic globally due to the rapid scale-up of the provision of anti-retroviral therapy (ART) through public health programmes. By the end of 2011, approximately 6.2 million people living with HIV/AIDS in sub-Saharan Africa were receiving ART, compared to only 100,000 in 2003 [1, 2]. Further efforts to reach the goal of universal access to treatment pose challenges, particularly in the current time of fiscal constraint [3].

Like other countries in the region, Malawi faces a generalized HIV epidemic, with an estimated prevalence of 11% in 15–49 year olds [4]. While only 13,000 people nationally were receiving treatment in 2004, by the end of 2011, 84% of all health facilities provided ART services, and over 440,000 had initiated ART with more than 320,000 people alive and retained in care [5]. Malawi has accomplished this through an efficient system that has substantial in-country political support matched with consistent external assistance [6–9]. The key features include a gradual increase in the number of sites offering anti-retrovirals; a focus on a single, generic, fixed-dose combination ART; treatment that is delivered free of charge; a standard protocol for starting therapy and for follow-up; and the use of a standard system for registration, monitoring and reporting of cases and outcomes [10].

An important part of the response in Malawi has been to decentralize the delivery of ART and related services, moving treatment from large, central district hospitals and tertiary care centres to primary health care settings with the consequence of increasing equity in access to the rural poor [11]. This is essential, as the Malawian Ministry of Health (MOH) attempts to double the number of people on ART from 250,000 in 2010 to 500,000 by 2015 [12]. DC has been found to be a safe and effective way to deliver care, with good levels of adherence and overall outcomes [13]. A key assumption of decentralization has been that it is cost-saving in particular for patients, both in terms of reducing foregone wages and lowering transport costs [14, 15]. These can be significant barriers to accessing care and maintaining continuity of care even where care and medications are free [16], and may explain in part why the poor have been found to have lower access to HIV testing and ART in several settings [17, 18].

This study contributes to our understanding of patient costs by surveying patients on ART in Malawi. By comparing costs borne by patients in a centralized programme to those in a decentralized programme of care, we add to what is known about the cost implications of scaling up [19]. In particular, we aim to quantify how much patients who access care close to where they live save in comparison to those who must travel to an urban area.

## Methods

This study is part of a broader cost-effectiveness analysis conducted by Dignitas International (DI), a Canadian academic non-governmental organization (NGO) that works in partnership with the Malawian MOH in the South-East Health Zone of Malawi. Research ethics approval was obtained from the Malawi National Health Sciences Research Committee. Written informed consent was obtained in the local language, Chichewa, from all study participants. All costs are reported in the local currency, in 2010 Malawian Kwacha (MKW) and US$2010, using a conversion rate of 1000 MKW to $6.50 [20].

### Study setting

In the Zomba District, publicly provided health care services are delivered through the Zomba Central Hospital (ZCH), which is located in the centre of the district, and 28 health centres scattered throughout the district under the direction of the Malawi MOH. Services and medications provided by the MOH are free of charge and without user fees. Mission facilities and NGOs also provide health care services, each with a formal service agreement with the MOH.

DI has been working with the MOH to deliver HIV/AIDS care in Zomba District since 2004. Initially, ART provision occurred with the opening of a specialist HIV/AIDS clinic at ZCH in October 2004. By 2007, over 250 patients per month were being enrolled on ART and during 2008 there were over 60,000 patient visits annually to the clinic. Beginning in January 2007, DI began to pilot the decentralization of ART provision to six rural health centres, meaning that patients that were initiated on treatment at ZCH could be followed and maintained on ART without needing to be seen at the main hospital. This was gradually expanded to 16 sites by April 2008. Soon after health providers at these centres were initiating patients on ART, with support from a DI staff who would visit sites every few weeks. By June 2009, more than 50% of patients on ART in the DI programme were followed at rural health centres. DI has enrolled over 18,000 people on ART as of the end of 2011, and patients can be seen at 24 rural health facilities in the district.

### Study design and data collection

To characterize patient costs borne by those in the centralized care (CC) programme compared to those in the decentralized care (DC) programme, we conducted a cross-sectional survey of patients. The survey was developed to focus on patient characteristics and costs associated with seeking care, particularly travel costs, out-of-pocket expenses and lost income from being absent from work. Patients in DC were also asked if they had been previously in CC and, if so, whether there was a difference in their costs. These questions were first pilot tested on 30 patients in CC and 30 in DC sites to ensure that the questions were clear and understandable. A single research assistant, who was fluent in Chichewa, recruited participants, obtained informed consent and administered the survey.

Participants were recruited by convenience sampling. DI maintains a list of patients on ART and while it may have been possible to randomly select participants, it was not technically feasible to contact patients between appointments. Recruitment occurred in the waiting areas of clinics with the assistance of local health clinic staff. Participants were provided with a 100 MKW ($0.65) honorarium for their time, or the equivalent of the cost of a snack or drink.

Participants were recruited at ZCH and five DC sites that are scattered through the district: Mayaka (41.5 km from Zomba), Pirimiti (25 km from Zomba), Domasi (16 km from Zomba), Thondwe (22 km from Zomba) and Chingale (30.5 km from Zomba).

These sites were chosen because they served the largest number of patients on ART and had the longest history of initiating and following patients on treatment. Patients who are seen at the five DC sites typically live in the immediate community that the clinic serves. Inclusion criteria were as follows: 15 years of age or older, being HIV positive and on ART for three months or more, followed at either ZCH or one of the five DC sites and able to provide informed consent. Given that many patients at ZCH utilize the facility simply because it is the closest health centre to where they live, and that we aimed to compare the experience of patients who lived in similar contexts, we excluded those who lived in the town of Zomba itself. All participants were interviewed during a three-week period in May 2010.

### Statistical analysis

Sample size was estimated based on the assumption of a mean total cost in CC of 300 MKW and in DC of 150 MKW, with a standard deviation of 300 MKW in each, using *α*= 0.05 and power of *β*=0.8. Differences between groups were compared using chi-squared tests for categorical variables and *t*-tests for continuous variables. We considered a *p*-value of 0.05 or less significant. Ordinary least squares were used to perform a regression analysis to identify significant predictors of reported total costs. Predictor variables included age and gender, as well as those that were significantly correlated to costs and not collinear with one another. Given that cost data was anticipated to be significantly skewed [21], when performing *t-*tests on costs between CC and DC, and in our regression analysis, we used non-parametric bootstrapping with 1000 replications [22, 23]. Data analysis was performed using Stata 12 (StataCorp, College Station, Texas).

## Results

### Characteristics of participants

We interviewed 120 patients who were receiving care through CC and 120 patients who were receiving care within DC. The latter were almost equally distributed across the five selected health centres: Mayaka (32 participants), Pirimiti (21 patients), Domasi (20 patients), Thondwe (25 patients) and Chingale (22 patients).

The characteristics of the participants are shown in Table 1. There were no significant differences between the groups in terms of gender and age. The majority of participants were female (77% CC and 85% DC) and the mean age was 39.6 years in CC and 38.0 years in DC. Significant differences in educational attainment were observed (*p*<0.001), with participants in CC being more likely to have attained a higher level of education than those in DC. Also, significant differences in occupation (*p*=0.03), mean monthly personal income (*p*=0.007) and mean monthly household income per household member (*p*=0.005) were seen between the groups. The most common occupation in both groups was subsistence farming, with a mean personal income of 3450 MKW ($22.43) per month. This represented a larger proportion of the DC group (53%), who were less likely to have higher income jobs such as paid employment, with a mean income of 5339 MKW ($34.70) per month, or operating a small-scale business, with a mean income of 12,726 MKW ($82.72) per month.

**Table 1:** Characteristics of participants

	Centralized care			Decentralized care			
		
	Males	Females	All	Males	Females	All	*p*
*N*	28 (23%)	92 (77%)	120	18 (15%)	102 (85%)	120	0.10
Age							
Mean (standard deviation)			39.6 (9.7)			38.0 (10.1)	0.20
20–29	3	15	18 (15%)	3	24	27 (23%)	0.440
30–39	11	37	48 (40%)	5	41	46 (38%)	
40–49	6	26	32 (27%)	5	20	25 (21%)	
50 +	8	14	22 (18%)	5	17	22 (18%)	
Educational attainment							0.001†
None	2	4	16 (13%)	3	34	37 (31%)	
Primary school	17	57	74 (62%)	13	64	77 (64%)	
Secondary school	9	20	29 (24%)	2	4	6 (5%)	
College/university	0	1	1 (1%)	0	0	0 (0%)	
Occupation							0.03†
Subsistence farming	11	32	43 (36%)	10	54	64 (53%)	
Piece-work	2	3	5 (4%)	1	5	6 (5%)	
Remittances	0	11	11 (9%)	1	7	8 (7%)	
Small-scale business	10	34	44 (37%)	5	31	36 (30%)	
Paid employment	5	12	17 (14%)	1	5	6 (5%)	
Mean monthly personal income	13,171 MKW ($85.61)	7695 MKW ($50.02)	8973 MKW ($58.32)	3956 MKW ($25.71)	4420 MKW ($28.73)	4351 MKW ($28.28)	0.007†
Mean monthly household income per household member	3408 MKW ($22.15)	2709 MKW ($17.61)	2872 MKW ($18.67)	1420 MKW ($9.23)	1640 MKW ($10.66)	1607 MKW ($10.45)	0.005†

† Significant difference between CC and DC.

### Amount of time spent seeking care

The amount of time participants reported in travel and at the clinic is summarized in Table 2. Those in CC and DC reported travel times that were very similar, with most taking 1–2 hours to reach the clinic from home (*p*=0.63). They spent a similar amount of time waiting at the clinic to be seen (*p*=0.63) and there was no significant difference between the total time spent seeking care (*p*=0.65).

**Table 2:** Time spent seeking care per clinic visit reported by participants in CC and DC

	Centralized care			Decentralized care			
		
	Males	Females	All†	Males	Females	All†	*p*
Travel time (one-way)							0.65
<1 hour	5	8	13 (11%)	7	12	19 (16%)	
1–2 hours	15	39	54 (45%)	8	42	50 (42%)	
2–3 hours	5	30	35 (29%)	2	29	31 (26%)	
>3 hours	3	15	18 (15%)	1	19	20 (17%)	
Mean time spent at clinic (SD)	4.12 (1.30) hours	3.82 (1.35) hours	3.89 (1.34) hours	3.30 (1.62) hours	3.88 (1.52) hours	3.80 (1.54) hours	0.63
Mean total time spent seeking care (SD)	7.04 (1.94) hours	7.33 (2.28) hours	7.26 (2.20) hours	5.54 (1.90) hours	7.41 (2.31) hours	7.13 (2.34) hours	0.65

† May not sum to 100% due to rounding errors.

### Costs associated with decentralized vs. centralized care

Participant answers to questions about costs are summarized in Table 3. The only significant differences in costs between the two groups occurred with travel-related expenses (*p*<0.001). This is related to the type of travel participants reported that they used. In CC, 60% of participants reported paying to use a mini-bus to reach the clinic, a major form of local transportation in Malawi. The remaining 40% reported travelling on foot or using a bicycle, not a recurrent cost. For those in DC, only 4% reported using a mini-bus and the remainder reported travelling on foot or by bicycle. Only one DC participant reported using a private vehicle. Of note, men in the DC programme (*n*=18) reported on average 0 MKW ($0) in travel costs, but this was not significantly different than women in the DC programme (*p*=0.134), given the large standard deviations and the relatively small numbers in each cell.

**Table 3:** Costs per clinic visit reported by participants in CC and DC

	Centralized care†			Decentralized care†			
			
	Males	Females	All	Males	Females	All	*p*
Travel costs (round-trip)	200 (241) MKW $1.30 ($1.57)	207 (277) MKW $1.35 ($1.80)	205 (268) MKW $1.33 ($1.74)	0 (0) MKW $0.00 ($0.00)	68 (189) MKW $0.44 ($1.23)	57 (176) MKW $0.37 ($1.14)	0.001‡
Additional costs	101 (82) MKW $0.66 ($0.53)	84 (110) MKW $0.55 ($0.71)	88 (104) MKW $0.57 ($0.68)	64 (66) MKW $0.42 ($0.43)	111 (172) MKW $0.72 ($1.12)	104 (161) MKW $0.68 ($1.05)	0.352
Income loss	257 (838) MKW $1.67 ($5.45)	51 (197) MKW $0.33 ($1.28)	99 (443) MKW $0.64 ($2.88)	67 (157) MKW $0.44 ($1.02)	66 (212) MKW $0.43 ($1.38)	66 (204) MKW $0.43 ($1.33)	0.419
Total costs associated with seeking care	558 (948) MKW $3.63 ($6.16)	343 (414) MKW $2.23 ($2.69)	392 (586) MKW $2.55 ($3.81)	131 (194) MKW $0.85 ($1.26)	244 (386) MKW $1.59 ($2.51)	227 (365) MKW $1.48 ($2.37)	0.002‡

† Mean (SD).

‡ Significant difference between CC and DC.

Approximately 66% of CC participants and 68% of DC participants reported having additional out-of-pocket costs. The vast majority reported that this was for food (100% of those in CC and 88% of those in DC). Only nine participants, all in the DC group, reported that they also had expenses related to medication, as some health centres that are run through a service agreement with the Christian Hospitals Association of Malawi charge small fees for cotrimoxazole prophylaxis. No significant difference was seen between the groups in the total amount of additional out-of-pocket costs reported (*p*=0.375).

In the CC group, 26% of participants reported that they miss work when seeking care, while in the DC group this was 32% (*p*=0.256). Approximately 9% of the CC group reported that they lost income as a result of seeking care, and in the DC group this was approximately 16% (*p*=0.118). Although mean personal and household incomes were different between groups, and participants reported requiring the same amount of time to seek care, the amount of income lost was not significantly different (*p*=0.419). This is likely due to the large variation seen in the reported amounts.

### Relationship between socio-economic variables and costs

Given that there were significant differences observed between the groups in terms of educational attainment, occupation, personal income and household income per person, we examined whether these were correlated with total costs (travel costs, additional costs and income lost summed together). Total costs were stratified into approximately equal categories (no costs, 0–100 MKW, 101–300 MKW and over 300 MKW). Higher educational attainment was significantly related to having higher travel costs (*p*=0.03). Occupation type was also significantly related to reported travel costs, although small numbers in certain cells made statistical testing difficult to interpret. An individual's monthly income was significantly related to their total reported costs (*p*<0.001).

We conducted ordinary least squares regression analysis using bootstrapping, with predictor variables being the type of care (DC vs. CC), individual monthly income, age and gender. Education, occupation type and monthly income per household member were collinear with individual monthly income, hence were not included in the model. The type of care (*p*=0.048), age (*p*=0.02) and income (*p*=0.082) were significant predictors of total costs, independent of one another. Gender was not a significant predictor of total costs (*p*=0.486). This model suggests that participants in CC may experience total costs per visit of 91 MKW ($0.59) (95% confidence intervals: 1–182 MKW) greater than DC participants, adjusting for other factors.

### Cost differences with transfer from centralized to decentralized care

From the 120 participants in DC, 39 reported that they had previously received HIV/AIDS care within the CC programme. The mean of their reported estimated total cost while in CC was 425 MKW ($2.76). For these same participants, their mean current reported total cost in DC was 241 MKW ($1.57). The difference, 184 MKW ($1.19), is close to the average difference in mean total costs seen between CC and DC participants of 165 MKW ($1.07). This reinforces that the difference found between groups is valid.

## Discussion

This study examined costs associated with seeking HIV/AIDS care through a survey of 240 patients on ART in southern Malawi, half receiving care in a district hospital and half receiving care at local health centres. We found that patients in CC had greater costs when seeking care than DC patients, mostly related to travel costs. This difference is likely driven by the mode of travel used, as the majority of CC participants reported taking a mini-bus to reach the clinic. This difference is not surprising as CC participants lived outside the area where ZCH was located. Receiving care in DC saves patients money with each visit, not only for the patient but also for their guardian, who typically accompanies a patient to each visit. If a patient has six visits in a year, the estimated annual difference is 1092 MKW ($7.10), based on a mean of 91 MKW per visit for patient and guardian per visit. Given that >90% of the population lives on less than $2 per day (PPP) [24], even this small amount is likely a substantial sum for these patients.

Our findings match evidence from other studies in Malawi of patients on ART. Living far from treatment centres and high transportation costs were anticipated to be barriers to care as Malawi began to scale-up ART [25]. Urban–rural inequity in access to treatment was identified soon after the national programme got underway, and transportation costs were hypothesized as a key factor [26]. In a study of tuberculosis patients who were HIV positive, Zachariah et al. found that low transportation costs were associated with an increased acceptance rate of ART [16]. Patients in other Malawian studies have reported that personal financial constraints are barriers to achieving high adherence to ART [27], specifically high transportation costs [28, 29]. In other contexts, transportation costs have also been identified as potential barriers to engaging patients in care before they start ART [30] and as likely drivers of cost once patients are engaged in care [31]. Addressing any and all costs borne by patients has been seen as important to reducing loss to follow-up rates [32], particularly for patients in remote areas [33].

Several limitations to this study should be noted. First, participants were selected based on convenience sampling and not randomly selected; hence, this may not be fully representative of all patients on ART. Second, participants were not matched based on their education, occupation or income. We found significant differences in these variables between groups, with CC participants being slightly wealthier, more educated and more likely to work in higher paid occupations. These characteristics were correlated with travel costs and hence may explain part of the difference seen. While programme type was independently associated with costs, age and income were as well, hence this limits our conclusion that CC participants experience higher transportation related costs. Third, our study is based on self-report and respondents may have under or over-estimated costs. Ideally, costs for transportation and time spent in transit and in clinic would have been directly observed. Collecting information on precisely where patients lived in relation to the clinic would have added to our study, as would information on what forms of travel were available to participants and what factors influenced their choice. Finally, with respect to generalizability, costs likely vary over time and this was not captured in our cross-sectional survey. Of note, transportation costs have likely risen since data was collected due to the increase in fuel costs in Malawi since 2010. Our results may not be generalizable to other parts of the country where travel options, travel costs, the quality of the health care facilities and the composition of the population may be different. It is important to note that DC and CC are not complete substitutes for one another and all national ART programmes will require both forms of care to achieve universal coverage [34].

## Conclusions

Even in a system of HIV/AIDS care where patients do not pay out-of-pocket to see clinicians or for medications, they still incur costs. We found that most costs are travel related and that those in DC incurred lower costs. This has important implications for poorer, less educated patients in rural areas, for whom these costs may be significant. Our results can be useful for cost-effectiveness analyses that take a societal perspective. Further work is required to better characterise such costs and how they relate to adherence, missed appointments and whether patients forego other necessary goods to pursue care. Future studies could be conducted on the impact of travel vouchers on defaulting from care and being lost to follow-up. More research on what motivates patients to seek care at a local health centre or to travel to a larger hospital in an urban area would contribute to our understanding of patient-borne costs and preferences.
